# Trends in Ransomware Attacks on US Hospitals, Clinics, and Other Health Care Delivery Organizations, 2016-2021

**DOI:** 10.1001/jamahealthforum.2022.4873

**Published:** 2022-12-29

**Authors:** Hannah T. Neprash, Claire C. McGlave, Dori A. Cross, Beth A. Virnig, Michael A. Puskarich, Jared D. Huling, Alan Z. Rozenshtein, Sayeh S. Nikpay

**Affiliations:** 1 University of Minnesota, School of Public Health, Minneapolis, Minnesota; 2 University of Florida, College of Public Health and Health Professions, Gainesville, Florida; 3 University of Minnesota, Medical School, Minneapolis, Minnesota; 4 University of Minnesota, Law School, Minneapolis, Minnesota

## Abstract

**Importance:**

Anecdotal evidence suggests that health care delivery organizations face a growing threat from ransomware attacks that are designed to disrupt care delivery and may consequently threaten patient outcomes.

**Objective:**

To quantify the frequency and characteristics of ransomware attacks on health care delivery organizations.

**Design, Setting, and Participants:**

This cohort study used data from the Tracking Healthcare Ransomware Events and Traits database to examine the number and characteristics of ransomware attacks on health care delivery organizations from 2016 to 2021. Logistic and negative binomial regression quantified changes over time in the characteristics of ransomware attacks that affected health care delivery organizations.

**Main Outcomes and Measures:**

Date of ransomware attack, public reporting of ransomware attacks, personal health information (PHI) exposure, status of encrypted/stolen data following the attack, type of health care delivery organization affected, and operational disruption during the ransomware attack.

**Results:**

From January 2016 to December 2021, 374 ransomware attacks on US health care delivery organizations exposed the PHI of nearly 42 million patients. From 2016 to 2021, the annual number of ransomware attacks more than doubled from 43 to 91. Almost half (166 [44.4%]) of ransomware attacks disrupted the delivery of health care, with common disruptions including electronic system downtime (156 [41.7%]), cancellations of scheduled care (38 [10.2%]), and ambulance diversion (16 [4.3%]). From 2016 to 2021, ransomware attacks on health care delivery organizations increasingly affected large organizations with multiple facilities (annual marginal effect [ME], 0.08; 95% CI, 0.05-0.10; *P* < .001), exposed the PHI of more patients (ME, 66 385.8; 95% CI, 3400.5-129 371.2; *P* = .04), were less likely to be restored from data backups (ME, −0.04; 95% CI, −0.06 to −0.01; *P* = .002), were more likely to exceed mandatory reporting timelines (ME, 0.06; 95% CI, 0.03-0.08; *P* < .001), and increasingly were associated with delays or cancellations of scheduled care (ME, 0.02; 95% CI, 0-0.05; *P* = .02).

**Conclusions and Relevance:**

This cohort study of ransomware attacks documented growth in their frequency and sophistication. Ransomware attacks disrupt care delivery and jeopardize information integrity. Current monitoring/reporting efforts provide limited information and could be expanded to potentially yield a more complete view of how this growing form of cybercrime affects the delivery of health care.

## Key Points

**Question:**

How frequently do health care delivery organizations experience ransomware attacks, and how have the characteristics of ransomware attacks changed over time?

**Findings:**

In this cohort study of 374 ransomware attacks, the annual number of ransomware attacks on health care delivery organizations more than doubled from 2016 to 2021, exposing the personal health information of nearly 42 million patients. During the study period, ransomware attacks exposed larger quantities of personal health information and grew more likely to affect large organizations with multiple facilities.

**Meaning:**

The study results suggest that ransomware attacks on health care delivery organizations are increasing in frequency and sophistication; disruptions to care during ransomware attacks may threaten patient safety and outcomes.

## Teaser

This cohort study examines the frequency and characteristics of ransomware attacks on health care delivery organizations.

## Introduction

As health care delivery organizations have increased their reliance on health information technology, they have also increased their exposure to new cybersecurity risks, such as ransomware attacks. Ransomware is a type of malicious software that prevents users from accessing their electronic systems and demands a ransom to restore access.^1,2^ Ransomware attacks are one cause of health care data breaches, which are becoming more common,^3,4,5^ and are increasingly attributed to external causes (ie, hacking) rather than internal negligence or malfeasance (ie, misplaced laptops or inappropriately accessed data).^6^ Unlike other data breaches, which often seek to steal data, ransomware attacks are purposefully designed to disrupt business operations, thereby motivating the attacked organization to make the demanded payment.

Although ransomware attacks have existed for years, the US Federal Bureau of Investigation (FBI) and other government entities warn that widespread use of ransomware attacks against health care delivery organizations coincides with the COVID-19 pandemic.^7,8,9^ While some prominent ransomware attacks on health care delivery organizations have received considerable media attention,^10,11,12^ to our knowledge, there is presently no systematic documentation of the extent and effect of ransomware attacks. News coverage of individual attacks suggests that ransomware attacks are substantially disruptive to care delivery, with reports of computers and electronic health records being disabled or encrypted,^13,14,15,16,17^ clinicians forced to document care using pen and paper,^13,17^ appointments and surgeries delayed or canceled,^11,14,16,18,19,20^ emergency departments forced to divert ambulances,^11,14,15,17,20^ and practice infrastructure so damaged that some practices have opted to close rather than try to restore systems.^21^ Such instances of operational disruptions to the delivery of health care have been followed by some positing that ransomware attacks on health care delivery organizations may impose a human cost in addition to a financial one by jeopardizing patient safety and outcomes.^22,23^ In this study, we used a database of ransomware attacks on health care delivery organizations to quantify and describe this growing phenomenon.

## Methods

To conduct this study, we created a data source called the Tracking Healthcare Ransomware Events and Traits (THREAT) database and reported findings from the database. The THREAT database combines proprietary data provided by HackNotice (a cybersecurity threat intelligence company that helps businesses identify and respond to attacks) with data from the US Department of Health and Human Services Office of Civil Rights (HHS OCR) Data Breach Portal. The latter contains publicly available information that is collected when Health Insurance Portability and Accountability Act–covered entities report breaches of protected health information (PHI), as mandated by the Health Information Technology for Economic and Clinical Health Act of 2009. This study followed the Strengthening the Reporting of Observational Studies in Epidemiology (STROBE) reporting guidelines. This study was determined to be exempt from review and informed consent by the University of Minnesota institutional review board (common rule, category 5).

### Identifying Ransomware Attacks on Health Care Delivery Organizations

The THREAT database began with every corporate cybersecurity breach within the HackNotice system. HackNotice populated this database by crawling search engines (ie, systematically querying certain terms) and web-scraping sources, such as publicly reported databases (ie, the HHS OCR Breach Portal and other state-based reporting), search engines, news outlets, trade publications, and forums on the dark web (ie, the part of the internet requiring the use of specialized encrypted browsing technology) on which hackers advertise stolen data for sale and describe the success of their exploits. Data fields recorded in this system included organization name, date of breach, type of breach (eg, ransomware, website defacement), a narrative description of the breach, and source documentation (eg, news, official reporting, dark web).

From the list of HackNotice cybersecurity breaches, we identified breaches that occurred between 2016 and 2021 for which the affected organization was a health care delivery organization operating in the US. This involved web searches for each affected company’s name and/or domain. Our definition of “health care delivery organization” was intentionally expansive, including hospitals, clinics, diagnostic laboratories, dental offices, substance use treatment centers, pharmacies, emergency medical services, and post–acute care facilities.

To determine whether each data breach involved a ransomware attack, we searched supplemental sources, including press releases issued by the attacked organization, public disclosures (ie, posted copies of form letters sent to patients whose PHI was exposed during the attack), local news reports, and health care trade press coverage. Data breaches were deemed ransomware attacks if supplemental sources included mention of “ransomware” or other keywords indicating a ransomware attack (eAppendix in the Supplement).

### Publicly Reported Exposure of PHI

To quantify the number of individuals whose PHI was exposed during a ransomware attack, we relied on publicly reported information from the HHS OCR Data Breach portal.^4^ We matched attacks in the THREAT database to the HHS OCR database manually using the covered entity’s name, state, and date of breach reporting (eAppendix in the Supplement). Organizations are statutorily required to notify HHS of a data breach and the number of individuals affected within 60 calendar days of breach discovery.^24^ This information is made publicly available on the HHS website when the reported breach affects the PHI of 500 or more individuals. We additionally quantified whether a ransomware attack remained unreported to HHS OCR and the number of days that elapsed from the attack date to the reporting date.

### Categorizing Health Care Delivery Organization Type and Attack Breadth

Having identified the ransomware attacks affecting health care delivery organizations in the HackNotice data, we searched the previously mentioned supplemental sources and added additional details to the THREAT database for every attack, including attack breadth and health care delivery organization type. Attack breadth was defined as whether the ransomware attack affected a single or multiple health care facilities. We also relied on supplemental data sources and the attacked organization’s web page to identify health care settings affected, categorizing delivery organizations as hospital, ambulatory surgery center, clinic, dental, mental/behavioral health, post–acute care, and other (eg, emergency medical services clinicians, plastic surgery centers, and infusion centers). eTable 1 in the Supplement provides additional detail on how we categorized health care delivery organization type and attack breadth.

### Operational Disruptions

We collected reports on the type and duration of operational disruptions that occurred during ransomware attacks on health care delivery organizations. Common operational disruptions included ambulance diversion, canceled appointments/surgeries, and electronic system downtime. When available, we cataloged the duration of any operational interruption, as measured in days. Since supplemental sources frequently referenced a date at which operations were fully restored, we calculated the operational disruption duration as the number of days that elapsed from the date of the ransomware attack (ie, typically the date of the ransomware attack discovery; ransomware actors frequently have access for weeks or months before using malware that hinders operations) to the date of restoration. eTables 1 to 4 in the Supplement provide additional details on how we quantified operational disruptions and sources used.

### Statistical Analysis

We calculated the annual frequency of ransomware attacks and descriptive statistics for characteristics of those attacks, including details of public reporting of PHI exposure, status of encrypted/stolen data, health care delivery organization type(s) affected, and operational disruptions. To quantify changes over time in binary outcome variables, we used logistic regression models with the year of the ransomware attack as a continuous variable. To obtain average annual marginal effects, we used the margins command in Stata, version 16.1 (StataCorp). For 2 count-based outcome variables (ie, count of individuals whose PHI was exposed and count of days during which care delivery was disrupted by the ransomware attack), we used negative binomial regression models with the year of the attack as a continuous variable. All regression analyses used Huber-White robust standard errors to assess statistical significance, which was defined as *P* < .05. Analyses were conducted from May 2022 to October 2022.

## Results

**Figure 1.**
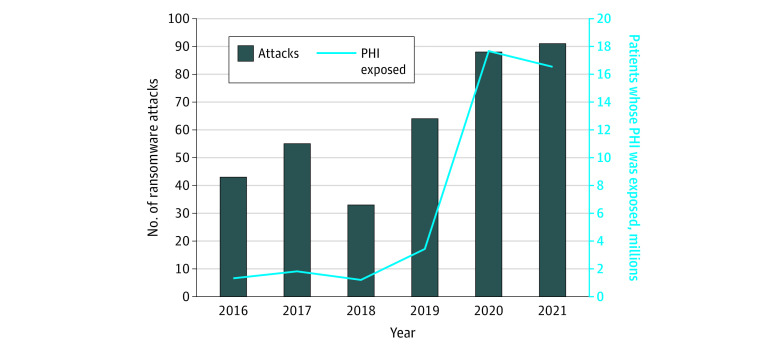
Number of Ransomware Attacks on US Hospitals, Clinics, and Other Health Care Service Delivery Organizations, 2016-2021 PHI indicates personal health information.

During the study period (2016-2021), we documented 374 ransomware attacks on health care delivery organizations that exposed the PHI of 41 987 751 million individuals (Figure 1). From 2016 to 2021, the annual number of ransomware attacks more than doubled, from 43 to 91. Personal health information exposure increased more than 11-fold, from approximately 1.3 million in 2016 to more than 16.5 million in 2021. A total of 84 ransomware attacks (22.5%) lacked information on PHI exposure, as they did not appear in the HHS OCR database (Table 1). Of the 290 ransomware attacks that were reported to HHS, most (203 [54.3% percent of all attacks]) were reported outside of the legislated reporting window of 60 days following the attack.

**Table 1.** Characteristics of Ransomware Attacks, 2016-2021

Characteristic	Ransomware	
	No. of attacks (n = 374)	Share of attacks, %
Public reporting		
Individuals whose PHI was exposed, mean	143 794	NA
Attack reported to HHS OCR	290	77.5
Attack reported late to HHS OCR	203	54.3
Status of encrypted/stolen data		
Data restored from backup	77	20.6
Some/all stolen PHI data made public	59	15.8
Characteristics of the affected health care delivery organization		
Attack affected multiple facilities	198	52.9
Type of health care delivery organization affected^a^		
Clinic	216	57.8
Hospital	82	21.9
Ambulatory surgical center	56	15.0
Mental/behavioral health	51	13.6
Dental	46	12.3
Post acute care	12	3.2
Other	80	21.4
Operational disruptions		
Disrupted care delivery	166	44.4
Disruption duration, mean d	15.8	NA
Known disruption with unknown duration	67	17.9
<1 wk	39	10.4
1-2 wk	28	7.5
2-4 wk	16	4.3
>4 wk	16	4.3
Type of disruption		
Ambulance diversion	16	4.3
Delays/cancellations in scheduled care	38	10.2
Electronic system (including EHR) downtime	156	41.7

Abbreviations: EHR, electronic health record; HHS OCR, Department of Health and Human Services Office of Civil Rights; NA, not applicable; PHI, personal health information.

^a^ Some categories (ie, health care delivery organization type, type of disruption) may sum to greater than 100%, as attacks could qualify for multiple categorical values. Duration of the operational disruption was only available for 99 ransomware attacks.

Across all 374 attacks, approximately 1 in 5 (20.6%) health care organizations were reportedly able to restore data from backups (Table 1). For 59 ransomware attacks (15.8%), there was evidence that ransomware actors had made some or all of the stolen PHI public, typically by posting it on dark web forums where stolen data are advertised for sale by including a subset of records.

Clinics (of all specialties) were the most common health care delivery organization type to experience a ransomware attack (Table 1), followed by hospitals, other delivery organization types, ambulatory surgical centers, mental/behavioral health organizations, dental practices, and post–acute care organizations. More than half (198 [52.9%]) of all ransomware attacks affected multiple facilities within the attacked organization.

**Figure 2.**
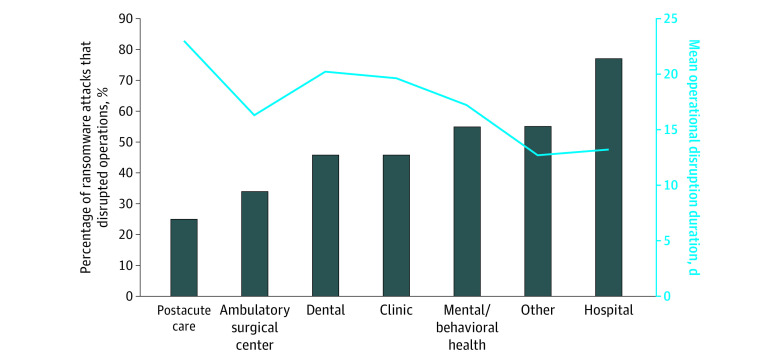
Operational Disruption Likelihood and Duration by Health Care Delivery Organization Type, 2016-2021 Ransomware attacks affecting multiple health care delivery organization types simultaneously appear in all relevant columns.

While all ransomware attacks are presumed to have some organizational effect in terms of activating system safeguards and leadership response, we documented evidence of care delivery disruptions during 166 ransomware attacks (44.4%) (Table 1). A total of 32 attacks (8.6%) were associated with a disruption exceeding 2 weeks. Types of care disruptions included electronic system downtime (156 [41.7%]), delays or cancellations of scheduled care (38 [10.2%]), and ambulance diversion (16 [4.3%]). Ransomware attack-induced operational disruptions varied by health care delivery organization type, with hospitals most likely to experience a disruption during a ransomware attack (Figure 2).

**Figure 3.**
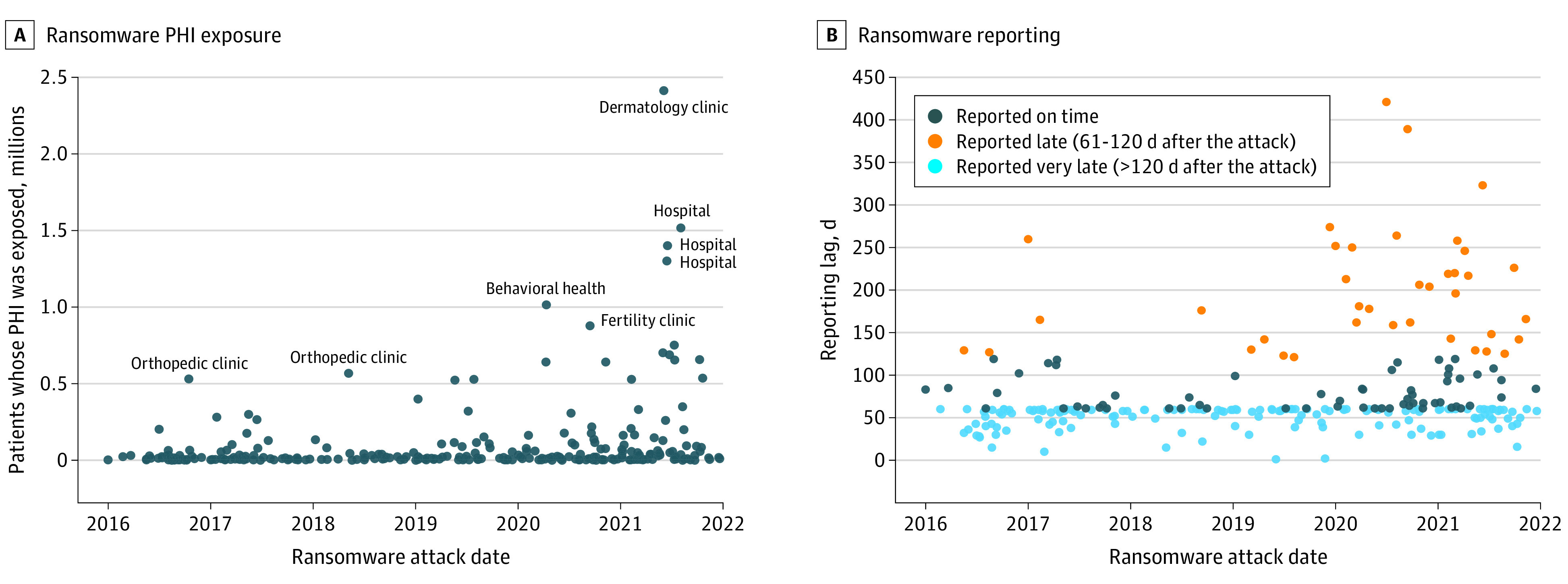
Personal Health Information (PHI) Exposure and Reporting During Ransomware Attacks Each dot represents a ransomware attack on a health care delivery organization. The 84 health care delivery organizations that experienced a ransomware attack but did not submit information to the US Department of Health and Human Services were excluded.

The characteristics of ransomware attacks on health care delivery organizations changed during the study period (Table 2; eTable 5 in the Supplement). With each year, ransomware attacks exposed the PHI of more patients (annual marginal effect [ME], 66 385.8; 95% CI, 3400.5-129 371.2; *P* = .04), a trend that was not limited to any single health care delivery organization type (Figure 3A). Over time, ransomware attacks were more likely to be reported late to the HHS OCR (ME, 0.06; 95% CI, 0.03-0.08; *P* < .001), and the number of attacks reported very late (ie, more than twice the statutory limit of 60 days from the attack) increased substantially in 2020 and 2021 (Figure 3B).

**Table 2.** Annual Change in Characteristics of Ransomware Attacks From 2016 to 2021

Characteristic	Ransomware attacks, No. (%)		Annual marginal effect (95% CI)^a^	*P* value
	2016	2021		
Public reporting				
Individuals whose PHI was exposed, mean	37 690	229 687	66 385.8 (3400.5 to 129 371.2)	.04
Attack reported to HHS OCR	35 (81.4)	72 (79.1)	−0.02 (−0.05 to 0)	.08
Attack reported late to HHS OCR	17 (39.5)	53 (58.2)	0.06 (0.03 to 0.08)	<.001
Status of encrypted/stolen data				
Data restored from backup	15 (34.9)	13 (14.4)	−0.04 (−0.06 to −0.01)	.002
Some/all stolen PHI made public	6 (14.0)	20 (22.2)	0.03 (0 to 0.06)	.02
Characteristics of the affected health care delivery organization				
Attack affected multiple facilities	18 (41.9)	70 (76.9)	0.08 (0.05 to 0.10)	<.001
Type of health care delivery organization affected				
Clinic	26 (60.5)	51 (56.0)	−0.02 (−0.05 to 0.01)	.13
Hospital	13 (30.2)	23 (25.3)	0.02 (0 to 0.05)	.12
Ambulatory surgical center	8 (18.6)	15 (16.5)	−0.00 (−0.02 to 0.02)	.89
Mental/behavioral health	3 (7.0)	18 (19.8)	0.04 (0.01 to 0.06)	.001
Dental	2 (4.7)	12 (13.2)	0.01 (−0.01 to 0.03)	.23
Post acute care	1 (2.3)	4 (4.4)	0.01 (−0.01 to 0.02)	.27
Other	8 (18.6)	22 (24.2)	0.02 (−0.01 to 0.05)	.11
Operational disruptions				
Disrupted care delivery	20 (46.5)	47 (51.7)	0.02 (−0.01 to 0.05)	.20
Disruption duration, mean, d	12.8 d	19.2 d	1.78 (−1.36 to 4.91)	.27
Type of disruption				
Ambulance diversion	1 (2.3)	7 (7.8)	0.01 (−0.00 to 0.03)	.09
Delays/cancellations in scheduled care	2 (4.7)	14 (15.6)	0.02 (0 to 0.05)	.02
Electronic system (including EHR) downtime	20 (46.5)	44 (48.9)	0.01 (−0.02 to 0.04)	.40

Abbreviations: EHR, electronic health record; HHS OCR, Department of Health and Human Services Office of Civil Rights; ME, marginal effect; PHI, personal health information.

^a^ Annual MEs were calculated using the margins command following logistic regression models that estimated the association between binary attack characteristic and year of attack (measured continuously). For count-variable attack characteristics, MEs were again calculated using the margins command following negative binomial regression models that estimated the association between characteristic and year of attack (measured continuously). Duration of the operational disruption was only available for 99 ransomware attacks.

From 2016 to 2021, the likelihood of health care organizations restoring ransomware-encrypted or stolen data from backups decreased (ME, −0.04; 95% CI, −0.06 to −0.01; *P* = .002), and more attacks were associated with some or all of the stolen PHI becoming public (ME, 0.03; 95% CI, 0-0.06; *P* = .02; Table 2; eTable 1 in the Supplement). During the study period, the probability that a ransomware attack affected multiple facilities simultaneously (ie, a larger organization) increased by eight percentage points annually (ME, 0.08; 95% CI, 0.05-0.10; *P* < .001). Mental/behavioral health care delivery organizations (ME, 0.04; 95% CI, 0.01-0.06; *P* = .001) were increasingly likely to experience ransomware attacks. While there was no statistically significant increase over time in operational disruptions overall, there was an increase in the likelihood that an attack was associated with delays or cancellations to scheduled care (ME, 0.02; 95% CI, 0.00-0.05; *P* = .02) and an increase (statistically significant only at *P* < .10) in the share of attacks that involved ambulance diversions (ME, 0.01; 95% CI, −0.0 to 0.03; *P* = .09).

## Discussion

In this cohort study conducted with data from 2016 to 2021, we documented 374 ransomware attacks on health care delivery organizations that affected the PHI of nearly 42 million patients. The growing number of attacks affecting large entities (those with multiple facilities) and the associated growth in PHI exposed (along with the diminishing likelihood that an organization could restore data from backups) suggest that ransomware attacks on health care delivery organizations have increased in sophistication as well as in frequency. To our knowledge, these findings represent the only census of ransomware attacks on health care delivery organizations. However, the study’s estimates of magnitude align with findings in the gray literature, and the trend over time is consistent with reports that ransomware actors increasingly targeted health care delivery organizations during the COVID-19 pandemic.^7,25,26^

Despite careful research, many of the statistics reported in this article are likely underestimates due to underreporting. For example, 1 in 5 ransomware attacks were not present in the HHS OCR database. This absence may be due to low PHI exposure (ie, attacks affecting fewer than 500 individuals need not appear in the HHS OCR public database) or, alternatively, because of confusion about whether ransomware attacks must be reported through official channels when they involve encryption, but not actual removal, of data from computer systems. Guidance from HHS states that when a ransomware attack occurs, Health Insurance Portability and Accountability Act–covered entities and their business associates need not report it if they can demonstrate a low probability that PHI has been exposed.^1^ Additionally, current reporting requirements lack either an enforcement mechanism or a penalty for noncompliance. Even when an entity reports an attack, there is no sanction for doing so outside of the legislated 60-day window, which may explain the high proportion (53.5%) of ransomware attacks with delayed reporting. Rather than health care organizations self-correcting as ransomware attacks become more common, we found an increase over time in the share of attacks that were reported late. Missing attacks and delayed reporting suggest opportunities for legislators who wish to strengthen data collection around cyberattacks, particularly ransomware, so as to shape an informed and well-targeted policy response.

Other information that is currently not tracked could potentially be incorporated into the existing reporting system. For example, policy makers could require the reporting of operational disruptions (eg, whether the health care delivery organization activated electronic health record downtime protocols and/or diverted ambulances) that occurred during a cyberattack. Administrative records, such as Medicare claims data, might also be used for similar purposes if the operational disruptions during ransomware attacks leave an identifiable signature. This approach might enable data collection without imposing additional reporting requirements on health care delivery organizations during an already challenging time. However, further research is needed to establish whether ransomware attacks create identifiable patterns in administrative data.

As is, this study’s findings regarding operational disruptions required individual research into each attack. Even with this constraint, we documented disruptions to care delivery during nearly half of all ransomware attacks, but the scope of the problem is likely larger. The most frequent disruption was to electronic systems, which frequently forced a switch to paper charting. Other documented disruptions included ambulance diversion and canceled appointments. These operational disruptions may harm patients, especially those experiencing emergencies and for whom timely treatment is crucial.^9^ Further study is needed to quantify an empirical association between ransomware attacks and patient outcomes.

Additional legislative activity concerns the ransom itself, with proposals to mandate disclosure (of ransom demands, whether a payment was made, and for what amount) and potentially even banning the payment of ransoms.^27,28^ The FBI strongly recommends that businesses not acquiesce to ransom demands in the event of a ransomware attacks, since complying with ransom demands incentivizes ransomware actors to continue targeting health care organizations. Going a step further, in 1 well-documented ransomware attack, law enforcement deliberately withheld the decryption key for nearly 3 weeks while planning an operation to disrupt the ransomware actors involved.^29^ To properly weigh law enforcement’s long-term deterrence goals against short-term patient safety goals, it is crucial to understand the association of ransomware attacks with patient safety and whether paying the ransom shortens the operational disruption.^22^ While it is intuitive to think that paying the ransom shortens the duration of any operational disruption, this is not necessarily the case; there are well-documented examples of follow-up ransom demands and nonfunctional decryption keys provided after ransom payments have been made.^30^ Additional ransom payment disclosure requirements would enable a better understanding of the potential tradeoff between financial cost and operational disruption duration.

Of equal if not more importance is identifying actions that health care delivery organizations can take to defend themselves effectively against ransomware attacks. Research suggests that health care delivery organizations are very susceptible to phishing emails that deceive insiders into giving access to hackers,^31^ and such emails are a common entry point for ransomware attacks. Existing cybersecurity recommendations require substantial time and money that many, but especially the most vulnerable rural and safety net health care delivery organizations, may not realistically have.^1,32^ Current estimates suggest that cybersecurity activities represent less than 10% of existing health system information technology budgets.^33^ To motivate increased investment, rigorous research is needed to identify actions that successfully thwart ransomware and other cybersecurity attacks on health care delivery organizations.

### Limitations

This study had several limitations. First, we likely omitted some ransomware attacks on health care delivery organizations. However, we believe that our database is the most comprehensive accounting of major health care ransomware attacks available for the period between 2016 and 2021. To be missing from the THREAT database, a ransomware attack would have needed to go unreported to HHS OCR, remain undetected by HackNotice web crawler surveillance and monitoring of dark web forums, and have received no press coverage in local news or health care trade publications. We believe this is most likely for ransomware attacks on smaller organizations, organizations in small geographic jurisdictions and/or organizations in states without mandated disclosure of data breaches, organizations without a hospital, and in scenarios in which the organization paid the demanded ransom quickly. The more likely scenario for omission of a ransomware attack from the THREAT database is misclassification. Events may have been ransomware attacks but only contained mention of malware or cyberattack, without discussion of any payment demand. To avoid false positives, these would not have been included in the THREAT database. Further research, including additional data sources, potentially in collaboration with the relevant federal entities (ie, the FBI, HHS OCR, the Health Sector Cybersecurity Coordination Center, and the Cybersecurity and Infrastructure Agency), is needed to validate this study’s findings.

Second, we have no insight into attempted but unsuccessful ransomware attacks (ie, a situation in which no one clicked on the phishing email link that would have compromised the system). Thus, we cannot comment on the traits of health care delivery organizations who avoid this type of cybercrime. Relatedly, we cannot attribute changes in the characteristics of ransomware attacks over time to changes in whom hackers target, the types of malware used, the market structure of health care delivery organizations (ie, as consolidation produces larger organizations, ransomware attacks are mechanically more likely to affect them), or to changes in organizational susceptibility to such cyberattacks and use of cybersecurity measures. Fourth, we likely underestimated the severity of operational disruptions and PHI exposure, as health care delivery organizations may try not to publicize these details. However, an increase in news coverage of ransomware attacks during the study period might bias us toward finding an increase in the sophistication of ransomware attacks. We noted that measures of attack sophistication and severity from multiple sources (ie, PHI exposure from the HHS OCR Data Breach Portal and operational disruptions covered by news/trade publications) yielded similar results. Fifth and finally, we could not say whether or how ransomware disruptions affect patients seeking care during an attack. Quantifying this remains a crucial area for future work.

## Conclusions

The results of this cohort study suggest that from 2016 to 2021, ransomware attacks on health care delivery organizations increased in frequency and sophistication. These attacks exposed PHI and frequently disrupted health care delivery, but further research is needed to more precisely understand the operational and clinical care consequences of these disruptions. As policy makers craft legislation aimed at countering the threat of ransomware attacks across multiple industries, we urge them to focus on the specific needs of health care delivery organizations, for which operational disruptions may carry substantial implications for the quality and safety of patient care.

## Notes

Supplement.
eAppendix.
**eTable 1.** Data Sources and Methodology for Ransomware Attack Characteristics **eTable 2.** Search Terms for Ransomware Attack Characteristics **eTable 3.** Ransomware Attack Count, by Category of Information **eTable 4.** Count of Attacks for which Each Source Provided Information **eTable 5.** Change (Presented as Odds Ratios and Incident Rate Ratios) in Characteristics of Ransomware Attacks, from 2016 to 2021
