# Errors in Figure 3B

**DOI:** 10.1001/jamahealthforum.2023.0001

**Published:** 2023-02-17

In the Original Investigation titled “Trends in Ransomware Attacks on US Hospitals, Clinics, and Other Health Care Delivery Organizations, 2016-2021,”^1^ there were errors in the labeling of the legend for panel B of Figure 3. “Reported on time” should be represented by light blue dots, not dark blue; “Reported late (61-120 d after the attack)” by dark blue dots, not orange; and “Reported very late (>120 d after the attack)” by orange dots, not light blue. This article was corrected online.
