# Supplementary material for: Trends in Ransomware Attacks on US Hospitals, Clinics, and Other Health Care Delivery Organizations, 2016-2021
Source: JAMA Health Forum. 2022 Dec 29;3(12):e224873. doi: 10.1001/jamahealthforum.2022.4873 (PMC9856685; doi:10.1001/jamahealthforum.2022.4873)
Supplement: Supplement. — eAppendix. eTable 1. Data Sources and Methodology for Ransomware Attack Characteristics eTable 2. Search Terms for Ransomware Attack Characteristics eTable 3. Ransomware Attack Count, by Category of Information eTable 4. Count of Attacks for which Each Source Provided Information eTable 5. Change (Presented as Odds Ratios and Incident Rate Ratios) in Characteristics of Ransomware Attacks, from 2016 to 2021 [file jamahealthforum-e224873-s001.pdf]

Supplemental Online Content

Neprash HT, McGlave CC, Cross DA, et al. Trends in ransomware attacks on US hospitals, clinics, and other health care delivery organizations, 2016-2021. *JAMA Health Forum*. 2022;3(12):e224873. doi:10.1001/jamahealthforum.2022.4873

eAppendix.

eTable 1. Data Sources and Methodology for Ransomware Attack Characteristics

eTable 2. Search Terms for Ransomware Attack Characteristics

eTable 3. Ransomware Attack Count, by Category of Information

eTable 4. Count of Attacks for which Each Source Provided Information

eTable 5. Change (Presented as Odds Ratios and Incident Rate Ratios) in Characteristics of Ransomware Attacks, from 2016 to 2021

This supplemental material has been provided by the authors to give readers additional information about their work.

© 2022 Neprash HT et al. *JAMA Health Forum.*

Process for Identifying Data Breaches that were Ransomware Attacks

To determine whether each healthcare provider data breach was specifically a ransomware attack, we searched supplemental sources, including press releases issued by the attacked organization, public disclosures (i.e., posted copies of form letters sent to patients whose PHI was exposed during the attack), local and national news reports, and healthcare trade press coverage. Data breaches were deemed ransomware attacks if sources included mention of the following keywords:

- "Ransomware"
- "Malware" AND "ransom" OR "payment" OR "payment demand" OR "extort%"
- "Cyberattack" AND "ransom" OR "payment" OR "payment demand" OR "extort%"

When supplemental sources mentioned malware or a cyberattack without explicitly referencing a payment demand or extortion attempt, the breach was not categorized as a ransomware attack. We further classified a data breach as ransomware if the HHS OCR Data Breach Portal archived database explicitly called the breach a "ransomware" attack in the Web_Description variable. This conservative approach was designed to minimize false positives and likely results in an undercount of true ransomware attacks on healthcare providers.

Process for Quantifying Characteristics of Ransomware Attacks

Beyond demonstrating the increasing frequency of ransomware attacks on healthcare providers, we also quantified the growing sophistication of those attacks. To measure changes in the characteristics of ransomware attacks, we focused on four categories: public reporting of attacks (i.e., number of individuals whose PHE was exposed, whether an attack was reported to HHS, and whether that attack was reported within the legislated 60-day window)), status of encrypted/stolen data (i.e., whether data were restored from backup, whether stolen PHI was made public in whole or part), provider type affected, and operational disruptions (i.e., whether care delivery was disrupted during the ransomware

© 2022 Neprash HT et al. *JAMA Health Forum.*

attack, for how long, and how). Table S1 includes additional detail on the sources for every ransomware attack characteristic and variable construction.

© 2022 Neprash HT et al. *JAMA Health Forum.*

eTable 1. Data Sources and Methodology for Ransomware Attack Characteristics

**Ransomware Attack Characteristic**	**Source**	**Description**
Public Reporting		
Individuals whose PHI was exposed, mean	HHS OCR Data Breach Portal	Ransomware attacks were individually matched to the HHS OCR Data Breach Portal, using name of the attacked organization and date of attack discovery. We used the 'Individuals Affected' field to quantify PHI exposure.
Attack reported to HHS OCR	HHS OCR Data Breach Portal	1 if the HHS OCR Data Breach Portal had a record for the ransomware attack; 0 if not.
Attack reported late to HHS OCR	HHS OCR Data Breach Portal	For ransomware attacks matched to the HHS OCR Data Breach Portal, we calculated the number of days elapsed from date of attack discovery to date of report. We classified as 'late' any attack reported more than 60 days after it was discovered. This is the mandatory reporting deadline imposed by the HITECH Act of 2009.
Status of Encrypted/Stolen Data		
Data restored from backup	Press releases, public disclosures, and news coverage	1 if source(s) stated that the organization restored affected systems from backup; 0 if not.
Some/all stolen PHI data made public	Press releases, public disclosures, and news coverage + HackNotice documentation of dark web data sales	1 if source(s) stated that some/all of stolen data were made publicly available via the internet or dark web; 0 if not.
Provider Type Affected		

© 2022 Neprash HT et al. *JAMA Health Forum.*

Attack affected multiple facilities	Press releases, public disclosures, and news coverage + health care provider organization websites	1 if one of the following is true:<br>(1) Press release/public disclosures/news coverage does not indicate an attack limited to a single facility AND the provider organization's website makes clear that they operate multiple facility locations.<br>(2) The press release/public disclosures/news coverage affirmatively indicates that the attack affected multiple facilities within the healthcare provider organization. For example, this would be true if news coverage mentions and EHR outage at the hospital and cancelled appointments associated outpatient clinics;<br>0 if sources indicate that only one health care providing facility was affected and/or the organization's website lists only one facility location.
Type of provider affected	Health care provider organization websites	Classification into the following provider types, relying on documentation provided on the healthcare organization's website: hospital, ambulatory surgical center, clinic, post-acute care, mental/behavioral health, dental, and other (e.g., ambulance services, clinical laboratory, pharmacy, infusion center, dialysis provider, EHR vendor, software used to deliver radiation therapy, urgent care centers).
Operational Disruptions		
Disrupted care delivery	Press releases, public disclosures, and news coverage	1 if press releases, public disclosures, news coverage or the HHS OCR Data Breach Portal 'Web Description' field indicated that one or multiple of the three operational disruptions that we measured (ambulance diversion, delayed/cancelled care, electronic system downtime) occurred as a result of the ransomware attack; 0 if not.
Disruption duration (days), mean	Press releases, public disclosures, and news coverage	For ransomware attacks with news coverage (or press release disclosure) of when disruptions ended (i.e., when electronic systems were restored), we calculated the number of days elapsed between discovery of the ransomware attack and restoration of functionality. If/when a time range was reported (e.g., two to three weeks), we conservatively chose the shortest reported

		duration, to avoid overestimating the duration of operational interruptions.
Type of disruption	Press releases, public disclosures, and news coverage	Operational disruptions were classified into one of three categories: (1) ambulance diversion: a hospital's decision to invoke diversion status, resulting in ambulances being re-directed to other facilities; (2) delays/cancellations in scheduled care; and (3) downtime for electronic systems involved in providing care (e.g., electronic health records, patient scheduling and communication platforms). Additional detail on specific search terms contained in Table S2.

Since the measurement of many attack characteristics involved identifying the presence of specific words or text strings within press releases, public disclosure letters, and news coverage, we additionally list search terms relevant to each attack characteristic:

© 2022 Neprash HT et al. *JAMA Health Forum.*

eTable 2. Search Terms for Ransomware Attack Characteristics

**Ransomware Attack Characteristic**	**Search Terms**
Status of Encrypted Data	
Data restored from backup	"backup", "back-up", "restore%"
Some/all stolen PHI data made public	"data dump", "posted", "leak%"
Operational Disruption	
Ambulance Diversion	"ambulance" + "diver%"
Delays/cancellations in scheduled care	"cancel%", "resched%","postpone"
Electronic system (including EHR) downtime*	"offline","downtime", "encrypted", "shut down" + "electronic", "paper chart%", "disrup%"

***When sources specified that the affected server(s) were not critical to the delivery of patient care (i.e., servers with digitized records from decades ago)

Process for Matching THREAT Attacks to the HHS OCR Breach Portal Database

The HHS OCR Breach Portal Database includes all data breaches that impacted more than 500 individuals. We searched the Breach Portal for the name of each facility in the THREAT database. If an entry matched the name, state, and rough timeline of an attack, it was considered a "match". If no corresponding report was found, we examined all reported breaches in the same state and within 60 days of the attack. If any data breaches appeared similar or were filed under names associated with the attacked facility (i.e., name of larger health system, or a "doing-business-as" (or DBA) alias) they were deemed a match. This process may have missed matches because many providers report attacks after the 60-day deadline. In addition, we searched the HHS OCR database for breach descriptions that included the word "ransomware". Any breach reports that represented ransomware attacks not present in the THREAT database were added and matched to the corresponding HHS breach report.

Reporting of Sources for Ransomware Attacks

© 2022 Neprash HT et al. *JAMA Health Forum.*

Without publishing the full THREAT database here (please contact the corresponding author if interested in access), we provide additional detail in Table S3 on overlap in coverage between our sources of information (i.e., HackNotice data, the HHS OCR Data Breach Portal database, and press releases/public disclosures/news coverage). Table S4 includes the count of ransomware attacks for which each source provided information. Please note that this count is not mutually exclusive; rather, the count of >1000 attack sources indicates that the vast majority of our 371 ransomware attacks had multiple sources of information.

© 2022 Neprash HT et al. *JAMA Health Forum.*

eTable 3. Ransomware Attack Count, by Category of Information

	Ransomware Attacks	
**Source(s) of Information**	**N=**	**%**
Found only in HHS data	3	0.8%
Found only in HackNotice data	0	0.0%
Found only in supplemental sources (i.e., press releases, public disclosure letters, news coverage)	20	5.3%
Found in HHS data and HackNotice data, but not in supplemental sources	16	4.3%
Found in HHS data and supplemental sources, but not in HackNotice	12	3.2%
Found in HackNotice data and supplemental sources, but not in HHS	64	17.1%
Found in all three (HHS, HackNotice, and supplemental sources)	259	69.3%
TOTAL	374	100.0%

© 2022 Neprash HT et al. *JAMA Health Forum.*

eTable 4. Count of Attacks for which Each Source Provided Information

Source	# of attacks using reports from this source
Organizational Press Releases	51
Public Reporting Databases	
HHS OCR Data Breach Portal	289
California	20
Delaware	1
Iowa	2
Maine	1
Maryland	1
Massachusetts	5
Montana	12
New Hampshire	16
Vermont	3
Washington	1
News Coverage	
Local News (city or state)	64
National News	6
Trade Publications	
HIPAA Journal	173
DataBreaches.net	143
Health IT Security	68
Becker's Hospital Review	65
Healthcare IT News	25
SC Mag	13
Bleeping Computer	11
Fierce Healthcare	10
SpamFighter	7
HC Innovation Group	6
NetSec.news	5
Careers Info Security	4
Info Risk Today	3
Becker's ASC Review	2
Krebs On Security	2

© 2022 Neprash HT et al. *JAMA Health Forum.*

eTable 5. Change (Presented as Odds Ratios and Incident Rate Ratios) in Characteristics of Ransomware Attacks, from 2016 to 2021

	Ransomware Attacks, N (%)			
	**2016**	**2021**	**OR or IRR (95% CI)**	**P Value**
Public Reporting				
Individuals whose PHI was exposed, mean	37,690	229,687	1.59 (1.30-1.94)	<0.001
Attack reported to HHS OCR	35 (81.4%)	72 (79.1%)	0.88 (0.76-1.02)	0.08
Attack reported late to HHS OCR	17 (39.5%)	53 (58.2%)	1.27 (1.12-1.44)	<0.001
Status of Encrypted/Stolen Data				
Data restored from backup	15 (34.9%)	13 (14.4%)	0.80 (0.69-0.92)	0.003
Some/all stolen PHI made public	6 (14.0%)	20 (22.2%)	1.27 (1.04-1.55)	0.02
Provider Type Affected				
Attack affected multiple facilities	18 (41.9%)	70 (76.9)	1.40 (1.23-1.59)	<0.001
Type of provider affected				
Clinic	26 (60.5%)	51 (56.0%)	0.91 (0.81-1.03)	0.13
Hospital	13 (30.2%)	23 (25.3)	1.13 (0.97-1.32)	0.12
Ambulatory Surgical Center	8 (18.6%)	15 (16.5%)	0.99 (0.83-1.17)	0.89
Mental/Behavioral Health	3 (7.0%)	18 (19.8%)	1.40 (1.14-1.71)	0.001
Dental	2 (4.7%)	12 (13.2%)	1.11 (0.93-1.33)	0.23
Post-Acute Care	1 (2.3%)	4 (4.4%)	1.26 (0.85-1.87)	0.25
Other	8 (18.6%)	22 (24.2)	1.13 (0.97-1.32)	0.11
Operational Disruptions				
Disrupted care delivery	20 (46.5%)	47 (51.7%)	1.08 (0.96-1.22)	0.21
Disruption duration (days), mean	12.8 days	19.2 days	1.12 (0.92-1.37)	0.27
Type of disruption				
Ambulance diversion	1 (2.3%)	7 (7.8%)	1.42 (0.98-2.07)	0.07
Delays/cancellations in scheduled care	2 (4.7%)	14 (15.6%)	1.32 (1.06-1.65)	0.02
Electronic system (including EHR) downtime	20 (46.5%)	44 (48.9)	1.05 (0.93-1.19)	0.41

SOURCE: Authors' analysis of the THREAT database, 2016-2021
NOTES: Odds ratios (OR) are calculated from logistic regression models estimating the association between binary attack characteristic and year of attack (measured continuously). Incident rate ratios (IRR) are calculated from negative binomial regression models estimating the association between count-variable attack characteristic and year of attack (measured continuously). Abbreviations: PHI, personal health information; OR, odds ratio; IRR, incidence rate ratio; HHS OCR, Department of Health and Human Services' Office of Civil Rights; EHR, electronic health record.

© 2022 Neprash HT et al. *JAMA Health Forum.*
